# Contrastive Coloring in Horses

**Published:** 1892-08

**Authors:** Horace J. Smith


### COMMUNICATIONS.

---

*To Editors Journal of Comparative Pathology and Veterinary Archives:*

If Dr. Billings will consult the November number of the JOURNAL he will find a corrected report of my remarks on Dr. Peters' report. It reads, as corrected, "We have found Dr. Billings right in certain matters, while Dr. Salmon is right in certain other matters," and not as quoted by him in his article in the last number of the JOURNAL. A. W. CLEMENT.

---

### CONTRASTIVE COLORING IN HORSES.

*To the Editors of the Journal of Comparative Medicine and Veterinary Archives.*

GENTLEMEN :—In America, Great Britain, France, and in Germany, I have found horse-buyers who were afraid to buy horses with white feet, lest the animals should be more likely to go lame than horses that do not have white feet.

In America and in Great Britain, I have heard the lines :

> "One white foot buy him,
> Two white feet try him,
> Three white feet be sly of him,
> Four white feet and a white nose,
> Cut off his head and throw it to the crows."

Experience seems to have taught the plain people to avoid a white footed horse. It remains for scientific men to establish the fact, or disprove the assertion, that white footed horses go lame more frequently than others not so colored.

I would ask also, do mules and asses have white feet—and if not, why not ?

Scientific men should also give a reason why contrastive color on horses, if there is any, should almost universally appear at the extremities, i. e., at the feet and on the nose.

Though not a medical man, I venture the following hypotheses:

Black "points" on a solid bay—that is, black feet, muzzle, mane and tail may be given by nature as *an ornamental coloring ;* and so with horses of other colors—their contrastive coloring at the extremities may be simply for ornament.

Where, however, we have white "points," is it not a defect in the arterial circulation of the fœtus—whereby there is an amount of arterial blood insufficient for the full nutriment of the extremities—this deficiency being evidenced by lack of coloring matter being deposited at the feet and also on the nose—so that, co-ordinated with this lack of coloring matter is also a congenital weakness of the muscular system ?

It is asserted that negro infants at birth are born as white (colorless) as the infants of the Caucasian race—if this is so, what process occurs when arterial blood (ærated through the lungs) is furnished whereby pigment is furnished to the skin ?

My hypotheses are given with modesty with the hope that explanations from a scientific person or body competent for such duty may be given.

Very respectfully,

HORACE J. SMITH.

